# Unmet need for family planning and factors associated among women living with HIV in Oromia regional state, Ethiopia

**DOI:** 10.1186/s12978-021-01280-y

**Published:** 2021-11-13

**Authors:** Dereje Bayissa Demissie, Gizachew Abdissa Bulto, Rose Mmusi-Phetoe

**Affiliations:** 1 Department of Health Studies, College of Human Science, University of South Africa, Regional Learning Office Ethiopia, Addis Ababa, Ethiopia; 2 St. Paul’s Hospital Millennium Medical College, Addis Ababa, Ethiopia; 3 Department of Midwifery, College of Medicine and Health Sciences, Ambo University, Ambo, Ethiopia; 4 Department of Public Health, College of Human Science, University of South Africa, Pretoria, South Africa

**Keywords:** Modern contraceptives, Family planning, Met need, Unmet needs, Women living with HIV, Contraceptivos modernos, planejamento familiarplanejamento familiar, necessidades atendidas, necessidades não atendidas, mulheres vivendo com HIV

## Abstract

**Objectives:**

The purpose of this study was to determine the prevalence of the unmet need and identifying factors associated with the unmet need among women of reproductive age living with HIV in Oromia regional state, Ethiopia. One critical component of both a full range of contraceptives and satisfying demand for family planning with HIV services all women living with HIV is the appropriate model for HIV therapy, HIV prevention, and care with family planning services in a resource-limiting area like Ethiopia.

**Methods:**

Health facility-based cross-sectional study design was conducted among women living with HIV attending ART clinics in the special zone of, Oromia regional state, by simple random sampling was used to select 654 respondents. Both bivariate and multivariable logistic regressions analysis was used to identify at adjusted odds ratio (AOR) with 95% CI in the final model.

**Result:**

The study assessed the magnitude of demand for family planning among HIV-infected women and established that the demand was 630 (96.3%), of which 100 (16%) of women of reproductive age living with HIV had unmet needs for family planning while attending monthly ART clinic drug refilling and follow up. This study identified that factors found to be associated with met needs for family planning among women of reproductive age living with HIV attending ART/PMTC were discussions with healthcare providers (AOR = 4.33, 95% CI 2.56–7.32), previous pregnancy (AOR = 3.07, 95% CI 1.84–5.12); future fertility desire (AOR = 2.15, 95% CI 1.31–3.51); having sexual partners (AOR = 5.26, 95% CI 1.79–15.5) and the number of the sexual partner (one) (AOR = 7.24, 95% CI 1.82–28.74) were identified independent predictors of met needs for family planning.

**Conclusion:**

The overall demand for family planning was 96% among the women living with HIV, and that 16% of women had an unmet need for family planning. The authors conducted a logistic regression and find various dependent variables that are associated with the met need for family planning services, such as having discussions with healthcare providers, having a partner and previous pregnancy; future fertility desire, the last pregnancy being intended. These results are interpreted to suggest that clear policy implications of family planning must be better integrated into ART clinics.

## Plain Language summary

The overall demand for family planning was 96% among the women living with HIV, and that 16% of women had an unmet need for family planning. The authors conducted a logistic regression and find various dependent variables that are associated with the met need for family planning services, such as having discussions with healthcare providers, having a partner and previous pregnancy; future fertility desire, the last pregnancy being intended. It was established that high met need demand for family planning among HIV-infected women. These results are interpreted to suggest that clear policy implications of family planning must be better integrated into ART clinics an important conclusion of this study result. Policymakers would be better considers the future developments of national guidelines/strategies and training modules of family planning and HIV would be integrated family planning services into facility-based care for women living with HIV should consider these identified factors to increase the availability of family planning among women of reproductive age living with HIV. After the implementation of the final strategic plan, the integration of family planning and HIV services should lead to an increase in the utilization of family planning, dual contraceptive methods, the need for family planning being met, prevent repeated unwanted pregnancy, and offer HIV services. This will ultimately improve the quality of life of reproductive-aged women, the community, and families at large. Policymakers would better establish women-centered integrated family planning with HIV service could facilitate that the met need demand for family planning services of reproductive age women living with HIV.

## RESUMO

**Objetivos:**

O objetivo deste estudo foi determinar a prevalência da necessidade não atendida e identificar os fatores associados à necessidade não atendida entre mulheres em idade reprodutiva vivendo com HIV no estado regional de Oromia, Etiópia. Um componente crítico de uma gama completa de anticoncepcionais e satisfazer a demanda de planejamento familiar com serviços de HIV para todas as mulheres que vivem com HIV é o modelo apropriado para terapia de HIV, prevenção do HIV e cuidados com serviços de planejamento familiar em uma área com recursos limitados como a Etiópia.

**Métodos:**

O desenho de um estudo transversal baseado em unidades de saúde foi conduzido entre mulheres vivendo com HIV atendidas em clínicas de TARV na zona especial do estado de Oromia Regional, por amostragem aleatória simples foi usada para selecionar 654 respondentes. As análises de regressão logística bivariada e multivariada foram utilizadas para identificar a odds ratio ajustada (AOR) com IC de 95% no modelo final.

**Resultado:**

o estudo avaliou a magnitude da demanda de planejamento familiar entre mulheres infectadas pelo HIV e estabeleceu que a demanda era de 630 (96,3%), das quais 100 (16%) das mulheres em idade reprodutiva vivendo com HIV tinham necessidades não atendidas de planejamento familiar enquanto comparecia ao reabastecimento e acompanhamento de medicamentos da clínica ART mensal. Este estudo identificou que os fatores encontrados associados ao atendimento das necessidades de planejamento familiar entre mulheres em idade reprodutiva vivendo com HIV em TARV / PMTC foram discussões com profissionais de saúde (AOR = 4,33, IC 95%: 2,56-7,32), gravidez anterior (AOR = 3.07, IC 95% 1.84–5.12); desejo futuro de fertilidade (AOR = 2.15, IC 95%: 1.31–3.51); ter parceiros sexuais (AOR = 5.26, IC 95% 1.79–15.5) e o número do parceiro sexual (um) (AOR = 7.24, IC 95% 1.82–28.74) foram preditores independentes identificados de necessidades atendidas de planejamento familiar.

**Conclusão:**

A demanda geral de planejamento familiar foi de 96% entre as mulheres que vivem com HIV e 16% das mulheres tinham uma necessidade não atendida de planejamento familiar. Os autores realizaram uma regressão logística e encontraram várias variáveis dependentes que estão associadas ao atendimento da necessidade de serviços de planejamento familiar, como discussões com profissionais de saúde, ter companheiro e gravidez anterior; desejo de fertilidade futura, pretendendo-se a última gravidez. Esses resultados são interpretados para sugerir que as implicações políticas claras do planejamento familiar devem ser melhor integradas às clínicas de TARV.

## Introduction

The World Health Organization (WHO) has called on developing countries to ensure that women living in HIV-infected areas have access to a range of contraceptives as well as safe pregnancy, especially for those who are at risk of developing HIV and other sexually transmitted diseases [1]. Three key principles guided United States government’s global health policy to meet the family planning demands of HIV-positive women are an emphasis on reproductive rights via voluntarism and informed choice, quality service provision through evidence-based programming, and the creation of partnerships [2].

Women with unmet need are those who are fecund and sexually active but are not using any method of contraception, and report not wanting any more children or wanting to delay the next child. The concept of unmet need points to the gap between women’s reproductive intentions and their contraceptive behavior [3].

In many areas of the world where HIV prevalence is high, rates of unintended pregnancy and unsafe abortions are high. Of all pregnancies worldwide in 2008, 41% were reported as unintended or unplanned, and approximately 50% of these ended in abortion [4].

In 2012 it was estimated that in sub-Saharan Africa, 53 million women who wanted to avoid pregnancy were not using any family planning method [5]. Thus, the unmet need for contraception among women living with HIV in sub-Saharan Africa is high, with 66–92% of women reporting not wanting another child (now or ever), but only 20–43% of them using contraception [6]*.* The prevalence of unmet needs for family planning thus remains unacceptably high among women in sub-Saharan Africa, including those living with HIV, even if they are involved in HIV treatment programs [5].

According to the ‘Ethiopia Demographic and Health Survey’ (EDHS) of 2016 indicator, 28.9% of currently married women in the Oromia Region have an unmet need for family planning services; 17.1% for spacing, 11.8% for limiting, and only 28.6% women have received family planning services. The total demand for family planning in Oromia Region was 57.55% [7]. However, the percentage of married women with unmet needs for family planning has been declining over time, from 37% in 2000 to 22% in 2016. At the same time, the proportion of married women using modern contraceptive methods has increased sharply from 8% in 2000 to 36% in 2016 [7].

Meeting the unmet needs for family planning in sub-Saharan Africa could make an important contribution to improving maternal health through early studies or initiatives. In 2008, the estimated maternal mortality ratio in sub-Saharan Africa was 596 per 100,000 live births, the contraceptive prevalence was 22%, and the proportion of maternal deaths averted by contraceptive use was estimated at 32%. In contrast, among low-and-middle-income countries as a group, the maternal mortality ratio was 273, the contraceptive prevalence was 63%, and 44% of maternal deaths were estimated to be averted by family planning use [8].

The potential for contraceptives to reduce unintended pregnancies among women living with HIV—thereby contributing to the Prevention of Mother-to-Child Transmission (PMTCT) and reduced maternal morbidity and mortality—does not seem to be adequately empirically investigated, especially in terms of practitioners’ involvement in enhancing this practice. According to a study conducted in Ethiopia on HIV and family planning service integration at Voluntary HIV counseling and testing (VCT) among married and other sexually active women, 40% were using contraceptives. Of women in current sexual unions, 17% had unmet needs for family planning and were having unprotected sex [9].

It is seemingly not possible to reach the 2020 targets for reducing new pediatric HIV infections in high disease-burdened countries unless the unmet need for family planning among women living with HIV is dealt with [10]*.* People with HIV often desire a child or more children, and 20–60% have an HIV-negative partner [10, 11]. In sub-Saharan Africa, women are the HIV-positive partner in about half of discordant partnerships [12]*.* The WHO thus recommends that people with HIV who have serodiscordant partners be given the option of immediately starting ART to reduce sexual transmission and facilitate safe conception. It will also contribute to preventing new HIV infections among men and women [13]. Programs that have succeeded in promoting condom use and providing HIV prevention and treatment services in this region have largely missed the opportunity to address the contraceptive needs of the key populations they serve. Therefore, the research statement for this study is “What the unmet need for family planning of women of reproductive age living with HIV attending healthcare facilities in Oromia Region, Ethiopia?”.

## Methods and materials

### Study setting

This study was conducted in Ethiopia at Oromia regional state surroundings Finfinne/Addis Ababa. Finfinne referring to the parts of the Oromia region just neighboring the national capital and the capital city was not included. Finfinne was the original name in Afan Oromo language and it’s the capital city of Oromia regional state as well. The study zone is served by 27 health centers, 13 of which provided both ART and family planning services. The remaining 14 health centers provide family planning and all other maternal neonatal child health services except ART services. The total number of people living with HIV enrolled at ART clinics in the zone was 9421, of which 2380 were women of reproductive age (Office Finfinne Special Zone 2018:6) [20].

### Sampling procedure

All health centres found in the Special Zone of Oromia Regional state that provide both ART and family planning services were identified and randomly selected by computer-generated methods to be included in the study (5 Health centres were selected from 13 health centres). Of total reproductive age women attending ART and FP in the zone (2380), 65.4% (1557) are attended at five randomly selected health centers.

A list of all Reproductive age women living with HIV randomly selected Health facility, age between 18 and 49 years of age, who had attended ART follow-up services for at least 6 months from randomly selected healthcare facilities were created. The prepared sampling frame was prepared and entered into SPSS version 23 by using their pre-ART registration number from the health management information system (HMIS) database. A simple random sampling technique by computer-generated samples was utilized at each health centre to select a total of 670 study respondents by proportionally allocated five randomly selected health centres, based on their total number of ART clients based settled criteria.

### Study design

A health facility-based cross-sectional study design was conducted among Reproductive age women living with HIV attending both ART and Family planning clinics.

### Sample size determination

The sample size was determined through a single population proportion formula by using a case study found in integrated sites in Ethiopia, where 40% of women were family planning users (P) [14]. By considering the design effect of 2, with correction formula since the total population was less than 10,000 (2380) and with a 5% non-response rate considered, the final sample size was 670 women living with HIV.

### Data collection

The questionnaire used for data collection was initially prepared in English, and translated to Afan Oromo, and back to English for language experts to confirm its consistency. The questions included in the questionnaire were adapted and prepared by reviewing different related literature and variables identified to be measured. The training was given to data collectors and supervisors by the primary researcher for 2 days. Data collectors cross-checked the pre-ART card numbers of women living with HIV who came to the ART clinic with sampled card numbers daily. Five trained data collectors collected data from women of reproductive age. The completed questionnaires were collected and checked daily for consistency and completeness by supervisors and the primary researcher. Data were collected using a pre-tested structured Afan Oromo version of the questionnaire. A pre-test of the questionnaire was done on 5% of the women living with HIV at Ambo health center, to identify any ambiguity, to confirm consistency in the questionnaire, to determine acceptability, and to make necessary corrections 1 week before the actual data collection process. The respondents were guided through a questionnaire and chart abstraction conducted at their health facility by trained data collectors.

### Operational definition

According to this demand for family planning consists of women with meet need for FP who are using a contraceptive method to achieve their reproductive goals; unmet need, consists of women with an apparent demand for FP who are not using contraception/family planning [3]. Two different models were fitted to investigate the factors predicting the met need demand for family planning.

### Methods of analysis

The returned questionnaires were checked for completeness, cleaned manually, coded and entered into EPI INFO 7.1.6 version, and then transferred to SPSS version 23 for further analysis. Frequencies, percentages, mean and standard deviation (SD) were used to summarise descriptive statistics of the data and text. Moreover, tables and graphs will be used for data presentation. Bivariate analysis was used primarily to check which variables have an individual association with the dependent variable. This cross-sectional study surveyed women living with HIV in Oromia, Ethiopia to determine whether they expressed met demand need for family planning evidence on the logistic regression modelling was undertaken to examine the net effects of a set of explanatory variables over the outcome variables and COR, which were adjusted for all other variables at 95% CIs. In this analysis, the outcome variables, the demand for family planning were dichotomized with “1” being needs met and “0” being an unmet need for family planning. Two different models were fitted to investigate the factors predicting the demand for family planning. Accordingly, the HL test for the following two models showed Chi-square p-values > 0.05, which proved the goodness-of-fit of the applied models for this study at p = 0.81 for the demand of family planning model. This study revealed that based on the stated criteria the factors that were identified through binary logistic regression were family monthly income; discussion with healthcare providers about family planning; previous pregnancy; future fertility desire; and the number of sexual partners. These identified variables were entered into multiple logistic regression analyses to identify independent predictors of met need demands for family planning. Variables that were found to have an association with the dependent variables were then entered into multiple logistic regressions to control the possible effect of confounders. Finally, the variables which have significant association were identified based on AOR, with a 95% CI and p-value to fit into the final regression model.

## Result

The complete response rate of this study was 654/670 (97.6%).

There were 654 respondents whose ages ranged between 18 and 49 years. The mean age of the respondents was 31.86 years with an SD of ± 6.0 years as reflected in Table 1.

**Table 1:** Demographic and socioeconomic characteristics of respondents in Oromia Region, Ethiopia 2018 (N = 654)

Demographic and social characteristics	Category	Frequency (%)	Cumulative (%)
Age in year (n = 654) Mean (SD): 31.86 (± 6.0)	18–25	96 (14.7)	14.7
	26–35	374 (57.2)	71.9
	36–49	184 (28.1)	100.0
Highest level of education	Never been school	245 (37.5)	37.5
	Primary	284 (43.4)	80.9
	Secondary	106 (16.2)	97.1
	College/University	19 (2.9)	100.0
Employment status	Government employee	59 (9.0)	9.0
	Merchant/Private work	239 (36.5)	45.6
	Housewife	256 (39.1)	84.7
	Farmers	55 (8.4)	93.1
	Unemployed	45 (6.9)	100.0
Marital status	Married	528 (80.7)	80.7
	Cohabit/living together	51 (7.8)	88.5
	Divorced/separated	46 (7.0)	95.5
	Widowed	22 (3.4)	98.9
	Single	7 (1.1)	100
Residence	Urban	518 (79.2)	79.2
	Rural	136 (20.8)	100.0

Table 1 presented the education status of the respondents which revealed that the literacy proportion was 409 (62.5%). The majority of study participant’s religious affiliation 474 (72.5%) belonged to the orthodox denomination,

Of the 609 (93.1%) employed respondents, 256 (39.1%) were housewives and only 59 (9.0%) were working for the public service sector. The family’s monthly income distribution among the respondents was assessed, and it was found that on average, the income was 1398.18 Ethiopian Birr (50$), and ranged from 100 to 5000. More than 357 (54.6%) respondents were earning less than 1201 Ethiopian birr (1$ = 27.84Birr) see in Table 1 for details.

### The magnitude of met need demand for family planning of women living with HIV (N = 654)

The met need demand for family planning was measured by the summation of those who had met their needs for family planning, and those who had unmet family planning needs among sexually active women of reproductive age.

**Fig. 1:**
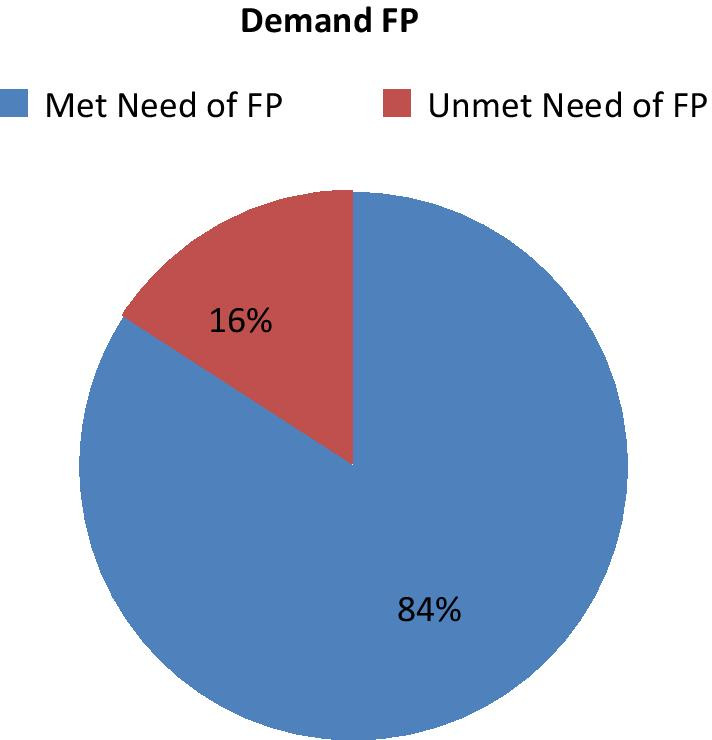
Magnitude of met need and unmet need demand for family planning among HIV-infected women 2018

The demand for family planning among women of reproductive age living with HIV was 630 (96.3%), at 95% CI of 94.8 to 97.7%, of which 530 (84%) had meet needs for family planning. The prevalence of unmet need for family planning was determined 100 (16%) of target populations had unmet needs for family planning while attending monthly ART drug refilling and follow-up programs at Health facilities which ranges from 13 to 19% at 95% CI see in Fig. 1.

### Factors associated with met need demand for family planning using multivariable logistic regression

This study revealed that based on the stated criteria the factors that were identified through binary logistic regression were family monthly income; discussion with healthcare providers about family planning; previous pregnancy; future fertility desire; and the number of sexual partners. These identified variables were entered into multiple logistic regression analyses to identify independent predictors of met need for family planning. Variables that were found to have an association with the dependent variables were then entered into multiple logistic regressions to control the possible effect of confounders. Finally, the variables which have significant association were identified based on AOR, with a 95% CI and p-value to fit into the final regression model.

According to the contents of Table 2, the respondents who had heard about family planning during ART follow-up had 35.8 times higher odds of meet need for FP as compared with had not heard family planning during follow up.

**Table 2:** Factors associated with met need for family planning at multivariable logistic regression (AOR, 95% CI) in Oromia, Ethiopia 2018

Factors associated with demand for family planning	Demand for family planning		AOR (95% CI)
	Met need for family planning	Unmet need for family planning	
Ever heard family planning services			
Yes	528 (80.7)	105 (16.1)	35.8 (7.48–171.4)***
No	2 (0.3)	19 (2.9)	
Discussed with healthcare			
Yes	470 (71.9)	73 (11.2)	4.326 (2.56–7.32)***
No	60 (9.2)	51 (7.8)	
Have partner			
Yes	521 (79.7)	103 (15.7)	5.26 (1.79–15.5)***
No	9 (1.4)	21 (3.2)	
Number of sexual partners			
None/zero	7 (1.1)	18 (2.8)	10.04 (3.49–28.84)***
One	507 (77.5)	94 (14.4)	7.24 (1.82–28.74)***
Two and above	16 (2.4)	12 (1.8)	
Last pregnancy wanted/timed			
Yes	443 (67.7)	71 (10.9)	3.07 (1.84–5.12)***
No	87 (13.3)	53 (8.1)	
Fertility desire			
Yes	256 (39.1)	68 (10.4)	2.15 (1.31–3.51)**
No	274 (41.9)	56 (8.6)	

*CI* confidence interval, *AOR* adjusted

***p < 0.001, **p < 0.01, *p < 0.05

Reproductive age women who had discussions with healthcare providers during follow up were 4.3 times higher odds of meet need for FP as compared with had not discussed FP needs during follow up. The study further reveals that those who had one or a single partner had 7.24 times higher odds of meet need for FP as compared with to those who had two and above sexual partners.

Respondents whose last pregnancy was intended had 3.07 times higher odds of meet need for family planning, and those who had fertility desire to have more children in the future were 2.15 times higher odds of meet need for family planning compared to those who had no intentions for pregnancy and had no fertility desire, respectively.

## Discussion

This study determined the magnitude of demand for family planning among HIV-infected women and established that the demand was 630 (96.3%), of which 530 (84%) had met the need for family planning, followed by 16% who had unmet needs in terms of family planning while attending monthly ART drug refilling with identified factors that affected met the need of family planning for reproductive-age women.

The socioeconomic characteristics of the respondents as summarised in Table 1 are not different from the socioeconomic profile of Ethiopia. For example, in the general population of the same region, Christian denominations dominate and represent 65% of the population, 79.2% resided in urban areas, and the largest ethnic group is Oromo, followed by Amhara which represent 64% of the population 7. The results are also similar in terms of the proportion of women who are currently married or living together with a partner (88.5% vs 65%) of women of ages 18–49 years in the region [7].

### The met need demand for family planning among HIV-infected women

One critical component of family planning and HIV integration that has significant positive health outcomes was to ensure that all women living with HIV have access to both a full range of contraceptives based on demand and safe pregnancy counselling [2]. This study determined the demand for family planning needs among women of reproductive age living with HIV was 96.3% with a 95% CI of 94.8 to 97.7% in the study area. This finding was supported by other studies which revealed that 92.6% of women living with HIV need family planning services [15].

On the other hand, these findings were higher than the general population of Ethiopia and the Oromia Region as evidenced by the demand for family planning is 58% [7: p. 20]. This might be due to the contribution of partnerships like PEPFAR and the United Nations Population Fund (UN family planning A) which supported pre-service training on family planning/HIV integration in the emergency surgical officer program, and the health extension worker program in Ethiopia [2]. There is evidence that suggests that a contribution of some family planning methods should increase demand for family planning. For instance, condom use increased from 5.7% pre-HIV to 71.7% post-HIV, with 89.6% of clients reporting regular use [16]. Therefore, this indicates the need for developing national guidelines/strategies and training for integrating family planning services into facility-based care for women living with HIV [17].

This study identified that 16% of women of reproductive age living with HIV had an unmet need for family planning while attending monthly ART drug refilling and follow-up programs. These findings concur with previous studies conducted in Kenya, which reported that 19% of clients who had been sexually active in the past month had an unmet need for family planning [18]. However, this was lower than the national general population, which indicated that the unmet need for family planning was 22% in Ethiopia [7: p. 20]. This result informs us that there is an unmet need for family planning among HIV-infected women, underlining a gap in the national PMTCT of HIV strategy.

This study identified factors that might be responsible for an increased met need demand for family planning during follow-up as being: a discussion with healthcare providers during follow-up; the number of sexual partners; last pregnancy planned; and fertility desire for more children in the future. The finding was supported by a study conducted in South Ethiopia [19], which revealed that factors that were significantly associated with the unmet need for family planning among women living with HIV were age, educational status, desired children, family planning not being used previously, not receiving family planning on the day of interview at HIV/AIDS care, and not being on ART. Therefore, future developments of national guidelines/strategies and training modules of family planning and HIV would be integrated family planning services into facility-based care for women living with HIV should consider these identified factors to increase the availability of family planning among women of reproductive age living with HIV.

### Limitation of the study

Though the problem of recall bias was minimized by conducting exit interviews; the current study is not free of social desirability bias in which some mothers may report the service as positive experiences while they are in the health facilities. As a strength, the study tried to cover a large number of health facilities including health centers and large sample size.

## Conclusion and recommendation

The overall demand for family planning was 96% among the women living with HIV, and that 16% of women had an unmet need for family planning. The authors conducted a logistic regression and find various dependent variables that are associated with the met need for family planning services, such as having discussions with healthcare providers, having a partner and previous pregnancy; future fertility desire, the last pregnancy being intended. It was established that high met need demand for family planning among HIV-infected women.

These results are interpreted to suggest that clear policy implications of family planning must be better integrated into ART clinics an important conclusion of this study result.

Policymakers would be better considers the future developments of national guidelines/strategies and training modules of family planning and HIV would be integrated family planning services into facility-based care for women living with HIV should consider these identified factors to increase the availability of family planning among women of reproductive age living with HIV.

Integrated family planning/HIV services contribute to the national family planning programs to provide full access to a variety of contraceptive methods so that couples and individuals can obtain the method that best suits their needs. The main contribution of the study to the level of health policy is as follows:

- Satisfy unmet needs and intention to use a method so that those wanting to use a method can do so.
- Provide quality counselling to improve the knowledge of reproductive-aged and empowered women by service providers on the integrated family planning/HIV services.
- Healthcare providers should be trained, equipped, and encouraged to take ownership of the implementation of the reproductive-aged women-centred integrated family planning /HIV strategic plan.
- After the implementation of the final strategic plan, the integration of family planning and HIV services should lead to an increase in the utilization of family planning, dual contraceptive methods, the need for family planning being met, prevent repeated unwanted pregnancy, and offer HIV services. This will ultimately improve the quality of life of reproductive-aged women, the community, and families at large by reducing unmet need for family planning.
- Policymakers would better establish women-centered integrated family planning with HIV service could facilitate that the met need for family planning services of reproductive age women living with HIV.

## Data Availability

Datasets used in the current study are available from the corresponding author upon reasonable request.

## Abbreviations

AIDS: Acquired immune deficiency syndrome
ART: Antiretroviral therapy
AOR: Adjusted odd ratio
CSA: Central Statistical Agency
CI: Confidence interval
COR: Crude odd ratio
DHS: Demographic Health Survey
EDHS: Ethiopian Demographic Health Survey
FHI: Family Health International
FMOH: Federal Ministry of Health
HIV: Human immuno-deficiency virus
IUDs: Intra uterine devices
OR: Odds ratio
ORHB: Oromia Regional Health Bureau
PMTCT: Prevention of Mother-to-Child Transmission
STIs: Sexually transmitted infections
UNAIDS: United Nations Programme on HIV/AIDS
UNICEF: United Nations International Children’s Emergency Fund
UNISA: University of South Africa
USAID: United States Agency for International Development
VCT: Voluntary HIV counseling and testing
WHO: World Health Organization
